# Old Molecule, New Chemistry: Exploring Silicon Phthalocyanines as Emerging N-Type Materials in Organic Electronics

**DOI:** 10.3390/ma12081334

**Published:** 2019-04-24

**Authors:** Nathan J. Yutronkie, Trevor M. Grant, Owen A. Melville, Benoît H. Lessard, Jaclyn L. Brusso

**Affiliations:** 1 Department of Chemistry and Biomolecular Sciences, University of Ottawa, 150 Louis Pasteur, Ottawa, ON K1N 6N5, Canada; nyutr055@uottawa.ca; 2 Department of Chemical and Biological Engineering, University of Ottawa, 161 Louis Pasteur, Ottawa, ON K1N 6N5, Canada; tgran079@uottawa.ca (T.M.G.); omelv065@uottawa.ca (O.A.M.)

**Keywords:** silicon phthalocyanines, n-type organic semiconductors, organic thin-film transistors

## Abstract

Efficient synthesis of silicon phthalocyanines (SiPc) eliminating the strenuous reaction conditions and hazardous reagents required by classical methods is described. Implementation into organic thin-film transistors (OTFTs) affords average electron field-effect mobility of 3.1 × 10^−3^ cm^2^ V^−1^ s^−1^ and threshold voltage of 25.6 V for all synthetic routes. These results demonstrate that our novel chemistry can lead to high performing SiPc-based n-type OTFTs.

## 1. Introduction

In recent years, research focused on the design of various organic architectures that enable electron transport in organic semiconductors (OSCs) has led to significant advances, not only in terms of OSC design but also the requirements for integration into organic electronics. In regard to the latter, to be viable for organic thin-film transistors (OTFTs), OSCs must ultimately possess high field-effect mobilities, robust environmental stability and ease of processability. While these efforts have led to impressive progress, the development of n-type OSCs still lags behind their p-type counterparts in terms of carrier mobility and air stability [1,2]. This may be attributed to the challenges associated with designing new n-type OSCs, in which the number of electron-deficient π-building blocks available is limited, and even fewer have been demonstrated to lead to high-mobilities [1]. Nonetheless, by employing motifs such as imides, carbonyls, *N*-heterocycles, cyanoethylene, and fullerenes, a library of both molecular and polymeric n-type OSCs has been achieved.

**Figure 1:**
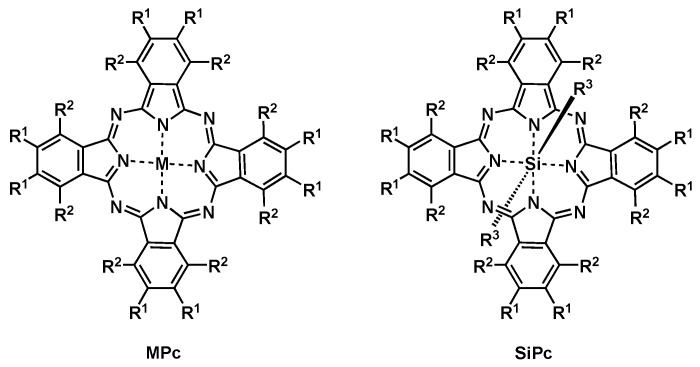
Divalent metal phthalocyanine (MPc) and tetravalent silicon phthalocyanine (SiPc) frameworks.

Phthalocyanines (Pcs; Figure 1), which are conjugated molecules composed of a tetramer of nitrogen-linked isoindole units, are ideal candidates for organic electronics. This is due in large part to their unique photophysical signatures and electrophysical properties, which are readily tunable through structural modification. Furthermore, they are known for their chemical robustness, high thermal resistance and low photodegradation [3,4]. Pcs often chelate a metal or metalloid through four M–N bonds, resulting in highly stable materials that have been explored for a variety of applications including their employment as active materials in organic electronics, predominantly as p-type materials [3,5,6,7,8,9,10]. With respect to their potential as n-type OSCs, only a handful of metal phthalocyanines (MPcs) have been shown to possess air stable n-type mobilities when implemented into OTFTs, with values on the order of 10^−1^ cm^2^V^−1^s^−1^ [9,11,12,13].

Replacing the central metal ion in MPcs with silicon affords tetravalent silicon phthalocyanines (SiPc), a subclass of phthalocyanines that have recently experienced an increase in interest with respect to integration into organic light emitting diodes [14,15,16], organic solar cells [17,18,19,20,21] and OTFTs [22], positioning SiPcs as excellent candidates as n-type OSCs. In addition to their inherent n-type mobility, a key advantage to employing SiPcs over divalent MPcs is the ability to modify the axial substituents (e.g., R^3^), thereby providing a facile avenue for functionalization. Considering that the two key factors that determine the performance of OSCs in organic electronics are the frontier molecular orbitals (in terms of both energy levels and electronic distribution), and molecular packing in the solid state, axial substitution in SiPcs enables modification, and ideally control, of the film morphology. To that end, preliminary studies on SiPcs axially functionalized with aromatic substituents revealed electron mobilities up to 10^−2^ cm^2^V^−1^s^−1^, demonstrating the influence of film morphology on device performance [22]. While axial substitution impacts molecular packing in the solid state, it has little effect on the energy levels of the frontier molecular orbitals. To that end, modifications to the R^1^ and R^2^ substituents about the Pc framework will not only influence the packing but also the energetics. This would therefore require appropriately substituted starting materials.

While MPcs are often prepared through the reaction of phthalonitrile with various metal precursors (e.g., MX_2_, where X = halide), this route is not effective for silicon-based derivatives as very low yields (i.e., less than 1%) [23] of SiPcCl_2_ are achieved upon the treatment of phthalonitrile with silicon tetrachloride (SiCl_4_) [24]. Other reported attempts to prepare SiPcCl_2_ involved the reaction of the disodium salt of phthalocyanine with SiCl_4_; however, no product was obtained through this methodology [25]. Alternatively, starting with diiminoisoindoline or o-cyanobenzamide in place of phthalonitrile affords enhanced yields of 71% and 35%, respectively [26]. Although this improved the yield of SiPcCl_2_, the preparation of such reagents proves problematic, especially of diiminoisoindoline (DIII). For example, the reported synthesis involves a constant flow of ammonia gas bubbling though a refluxing solution of phthalonitrile and sodium methoxide [26]. In addition to the harsh reaction conditions, the isolation of DIII of high purity is difficult to achieve, which may be attributed to many factors including its high solubility in a number of solvents, its tendency to cyclize upon dissolution and its sensitivity towards heat. This therefore limits the methods available for purification. As a result, DIII is often used in its crude form in most cases, or used directly in a one-pot synthesis of SiPcCl_2_ [27]. In order to tap into the potential of SiPcs, we sought to explore and develop new synthetic protocols to facilitate advancements within this exciting class of n-type OSCs. To that end, we herein present effective and alternative approaches to the classical preparative route for SiPcCl_2_, eliminating the undesired expenses attributed to the strenuous reaction conditions required and the hazardous reagents employed. Furthermore, integration of the resulting SiPc derivatives into high performing n-type OTFTs is presented, exhibiting the highest electron field-effect mobilities (µ_e_) reported for silicon phthalocyanine-based OTFTs. 

## 2. Results and Discussion

**Scheme 1:**
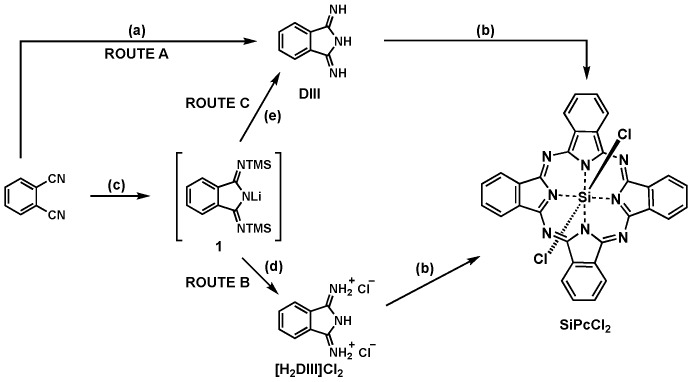
Synthetic routes in the preparation of SiPcCl_2_. Reagents and conditions: (**a**) (i) Na, MeOH, (ii) NH_3(g)_, Δ; (**b**) SiCl_4_, Quinoline, Δ; (**c**) LiN(TMS)_2_·Et_2_O, Toluene; (**d**) HCl_(g)_; (**e**) (i) TMSCl, (ii) silica gel.

As mentioned, SiPcs can be isolated following Route A (Scheme 1); however, the conditions required are not appealing for large scale production due to the safety precautions necessary when dealing with corrosive gases and highly reducing sodium metal. To achieve a more versatile and less hazardous preparative method, we sought to eliminate the necessity of both sodium metal and ammonia gas in the preparation of DIII. To that end, utilization of lithium bis(trimethylsilyl)amide etherate (LiN(TMS)_2_·Et_2_O) offers several advantages over the reagents required for Route A. First and foremost, LiN(TMS)_2_·Et_2_O is a solid tolerant to atmospheric conditions, rendering it easier to handle and quantify than gaseous reagents such as ammonia. Not only does the use of LiN(TMS)_2_·Et_2_O eliminate the need for ammonia gas, but it also affords the necessary basicity without the use of sodium metal, thereby affording a preparative route that removes the use of toxic gases (ammonia) and highly reactive reagents (sodium metal). As outlined in Scheme 1, treatment of phthalonitrile with LiN(TMS)_2_·Et_2_O affords the anionic intermediate 1. Initial attempts to quench with either water or methanol led to various intractable by-products, likely due to decomposition of the desired product. Alternatively, quenching the anionic intermediate with gaseous hydrochloric acid afforded the dihydrogen chloride salt [H_2_DIII]Cl_2_ (Route B), which precipitates out of solution as a powdery yellow solid. Attempts to deprotonate [H_2_DIII]Cl_2_ to afford DIII were unsuccessful; however, preparation of SiPcCl_2_ could be achieved by reacting [H_2_DIII]Cl_2_ directly with SiCl_4_ in refluxing quinoline.

Although SiPcCl_2_ can be synthesized via Route B in 21% yield, employing gaseous hydrochloric acid to isolate [H_2_DIII]Cl_2_ does not eliminate the use of corrosive gases. Alternatively, quenching 1 with chlorotrimethylsilane (TMSCl) affords the silylated DIII intermediate, while the LiCl by-product precipitates out of solution (Route C). Protonation and purification can be achieved upon passing the resulting brown solution through silica gel, affording DIII as a pale orange solid. Subsequent treatment of the DIII with SiCl_4_ in refluxing quinoline affords SiPcCl_2_ as a purplish blue matte solid in 51% yield. Via Route C, we have therefore established a safer alternative to that previously published, as we have not only eliminated the necessity for highly reactive reagents and corrosive gases, but we have also ascertained a method in which quantifiable amounts of each reagent necessary can be used. Overall, Route C offers the best alternative in terms of yield and purity of the final product and, through the use of LiN(TMS)_2_·Et_2_O in place of sodium metal and ammonia gas, paves the way for the development of additional derivatives that would otherwise not be possible with the previous synthetic routes.

**Figure 2:**
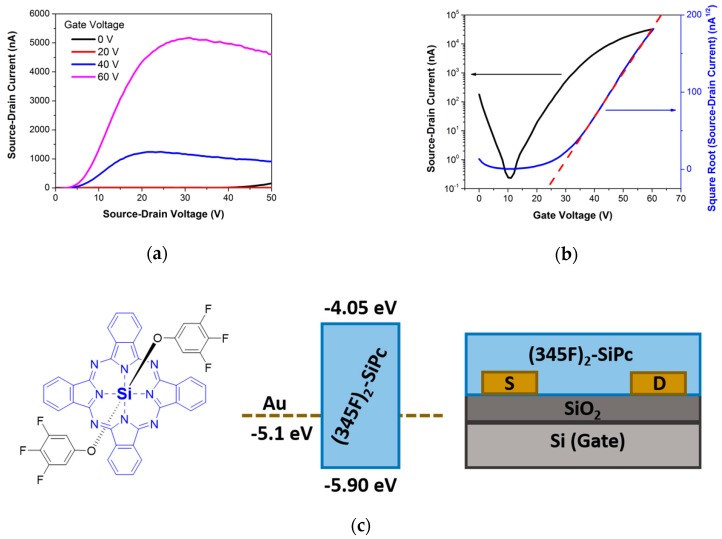
Characteristic output curve (**a**) and transfer curve (**b**) for bis(3,4,5-trifluorophenoxy) silicon phthalocyanine ((345F)_2_-SiPc) bottom-gate, bottom-contact (BGBC) organic thin-film devices with L = 5 um. (**c**) is the chemical structure of ((345F)_2_-SiPc), the respective highest occupied molecular orbital (HOMO) and lowest unoccupied molecular orbital (LUMO) levels of (345F)_2_-SiPc and a diagram of the BGBC organic thin-film transistor (OTFT) structure (from left to right) [28].

To test the efficacy of the various routes to prepare SiPcCl_2_, and to ensure the quality of the materials is maintained, samples from routes A–C were functionalized at the axial position with 3,4,5-trifluorophenoxy substituents and implemented into OTFTs. In that regard, bis(3,4,5-trifluorophenoxy) silicon phthalocyanine ((345F)_2_-SiPc) was synthesized from SiPcCl_2_ according to the literature [28], employing SiPcCl_2_ from Routes A, B and C. In all cases, the (345F)_2_-SiPc was purified by train sublimation and the products were obtained in similar yields with similar purities. UV–Vis absorption spectroscopy revealed no difference between all the (345F)_2_-SiPc derivatives in this study (Figure S1) and those reported in the literature [28]. (345F)_2_-SiPc has previously been reported to have a good thermal stability (decomposition temperature of >400 °C), significant pi–pi interaction by single crystal X-ray diffraction and a highest occupied molecular orbital (HOMO) level of −5.90 eV and a respective lowest unoccupied molecular orbital energy level of −4.05 eV (Figure 2c) [28]. Organic thin-film transistors (OTFTs) were therefore fabricated using (345F)_2_-SiPc (from Routes A, B and C) by thermal evaporation in a bottom-contact, bottom-gate configuration. Regardless of the synthetic route for the preparation of (345F)_2_-SiPc, an average electron field-effect mobility (µ_e_) of 3.1 × 10^−3^ cm^2^ V^−1^ s^−1^ and threshold voltage (V_T_) of 25.6 V were obtained. Although modest, this performance is superior to previously reported silicon-based phthalocyanine OTFT devices fabricated under similar conditions using bis(benzoate) silicon phthalocyanine as the semiconductor, where an average µ_e_ of 4.9 × 10^−4^ cm^2^ V^−1^ s^−1^ and V_T_ of 25.9 V were obtained [22]. For (345F)_2_-SiPc, performance varied over 75 devices with an average electron field-effect mobility of 3.1 × 10^−3^ cm^2^ V^−1^ s^−1^ and threshold voltage of 25.6 V, an on/off current ratio (I_on/off_) ranging between 10^3^ and 10^5^ and a maximum µ_e_ of 1.64 × 10^−2^ cm^2^ V^−1^ s^−1^ for all synthetic routes. Characteristic output and transfer curves for (345F)_2_-SiPc devices are shown in Figure 2. We have previously shown that when integrated into bottom-gate, bottom-contact OTFTs, (345F)_2_-SiPc and other SiPc derivatives experienced significant variations in performance as a function of channel lengths due to substantial contact resistance (as apparent in Figure 2a) [29]. Current efforts involve device engineering, including electrode material selection as a way to reduce contact resistance and amplify n-type performance.

## 3. Conclusions

In closing, we have demonstrated an efficient and effective method for the preparation of SiPcCl_2_ and, upon axial substitution, have confirmed their n-type mobilities, which are the highest reported to date for SiPc-based OTFTs. Having developed the synthetic methodology to isolate SiPcCl_2_ using less toxic, less corrosive, non-gaseous reagents, we can now begin to explore other derivatives with modified molecular frameworks. This therefore provides an avenue to control the energy levels of the frontier MOs, enabling the development of air stable n-type OSCs, in synergy with the combined morphological control through axial substitution. Such studies are currently underway.

## 4. Materials and Methods

### 4.1. General Methods and Procedures

The reagents phthalonitrile (TCI, Philadelphia, PA, USA), trimethylsilyl trifluoromethanesulfonate (Synquest, Alachua, FL, USA), chlorotrimethylsilane (Acros, Fair Lawn, NJ, USA), silicon tetrachloride (Sigma, Sheboygan, WI, USA) and 1,3-diiminoisoindoline (TCI, Philadelphia, PA, USA) were obtained commercially and used as received. Lithium bis(trimethylsilylamide) etherate [30], HCl gas [31] and bis(3,4,5-trifluorophenoxy) silicon phthalocyanine [32] were prepared as outlined in literature. All solvents were ACS grade; dry solvents were obtained by passing through activated alumina on a J.C. Meyer solvent purification system. Quinoline (Oakwood, Estill, SC, USA) was distilled over zinc dust. All reactions were performed under an atmosphere of dry nitrogen. NMR spectra were run in either DMSO-d_6_ or MeCN-d_3_ solutions at room temperature on a Bruker Avance 300 or 400 MHz spectrometer (Billerica, MA, USA, Figures S2–S4). ^19^F-NMR spectra were referenced to trifluoroacetic acid at −76.55 ppm (Figure S5). IR spectra of solid samples were recorded on an Agilent Technologies Cary 630 FT-IR spectrometer (Cary, NC, USA).

#### 4.1.1. Preparation of SiPcCl_2_ (via Route A)

Reaction was performed analogous to previously reported literature starting with 1 g of commercial diiminoisoindoline [27]. Crude yield 0.76 g (1.2 mmol, 73%). Samples were characterized spectroscopically and compared to references in literature [33]. HR-MS (EI) for C_32_H_16_Cl_2_N_8_Si [M]^+^: Calcd, 610.0644; Found, 610.0635.

#### 4.1.2. Preparation of 2,3-Dihydro-1H-Isoindole-1,3-Bis(Iminium) Bis(Trifluoromethanesulfonate) [H_2_DIII][OTf]_2_ (via Route B)

A solution of lithium bis(trimethylsilyl)amide etherate (4.7 g, 19.5 mmol) in dry toluene (200 mL) was added dropwise to a stirring tolyl solution (150 mL) of phthalonitrile (2.5 g; 19.5 mmol) and subsequently stirred overnight. Hydrochloric acid was bubbled into the mixture for 25 minutes. The resulting green slurry was stirred for 30 min, followed by filtration in vacuo and washed thrice with MeCN to afford the green solid of [H_2_DIII]Cl_2_. IR ν_max_ = 3120 (br, w), 2672 (br, m), 2545 (br, m), 2347 (br, w), 1671 (s), 1601 (m), 1527 (m), 1475 (m), 1463 (m), 1353 (m), 1318 (m), 1276 (m), 1179 (m), 1086 (w), 1043 (s), 912 (w), 846 (s), 826 (s, br), 787 (s), 746 (m), 728 (m), 690 (s). This material is essentially insoluble in organic solvents; its identity was determined by metathesis to the more soluble triflate salt by the following procedure: Trimethylsilyl trifluoromthanesulfonate (10.6 mL, 58.5 mmol) was added to a slurry of [H_2_DIII]Cl_2_ in dry MeCN (25 mL) and stirred for 1 h. The green slurry was filtered in vacuo and washed thrice with DCE to afford a green solid of [H_2_DIII][OTf]_2_. Yield 4.8441 g (10.9 mmol, 56%). ^1^H-NMR (δ, CD_3_CN, RT, 300 MHz): 8.36 (m, 2H), 8.11 (m, 2H). ^19^F-NMR (δ, CD_3_CN, RT, 300 MHz): −79.20 (s, 6F). IR ν_max_ = 3252 (w), 3109 (w), 2967 (m, br), 1678 (m), 1606 (w), 1478 (w), 1467 (w), 1353 (w), 1300 (m), 1284 (m), 1271 (m), 1236 (m), 1217 (s), 1192 (s), 1167 (s), 1091 (w), 1077 (m), 1033 (s), 1022 (s), 978 (w), 868 (m), 840 (m), 783 (m), 764 (m), 748 (m), 697 (m).

#### 4.1.3. Preparation of Silicon Phthalocyanine Dichloride (via Route B)

A solution of lithium bis(trimethylsilyl)amide etherate (4.7 g, 19.5 mmol) in dry toluene (200 mL) was added dropwise to a stirring solution of phthalonitrile (2.5 g; 19.5 mmol). After 5 h, the reaction mixture was cooled in an ice bath and hydrochloric acid was bubbled into the mixture for 25 min. The resulting green slurry was stirred for 30 min followed by filtration in vacuo and washed thrice with MeCN. To the filtered solid, dry quinoline (75 mL) and silicon tetrachloride (3.2 mL, 28.1 mmol) were added. The stirring slurry was heated to 180 °C for 4 h producing a visible red microcrystalline solid. The solid was filtered and washed with benzene, followed by methanol and acetone, respectively. Crude yield 0.63 g (1.0 mmol, 21%). Samples were characterized spectroscopically and compared to references in literature [33]. HR-MS (EI) for C_32_H_16_Cl_2_N_8_Si [M]^+^: Calcd, 610.0644; Found, 610.0632.

#### 4.1.4. Preparation of DIII (via Route C)

A solution of lithium bis(trimethylsilyl)amide etherate (4.7 g, 19.5 mmol) in toluene (200 mL) was added dropwise to a stirring tolyl solution (150 mL) of phthalonitrile (2.5 g; 19.5 mmol) and subsequently stirred overnight. Chlorotrimethylsilane (2.2 mL, 19.5 mmol) was added to the solution and refluxed for an additional 24 h. Lithium chloride was removed through hot filtration of the mixture in vacuo. The brown filtrate was passed through a column of silica and washed with DCM and EtOAc, respectively. The desired product was collected with a 25% MeOH in EtOAc solution (v/v). The solvent of the filtrate was removed in vacuo and the resulting brown solid was washed with ether, DCM, and EtOAc consecutively, resulting in a light orange solid. Yield 1.23 g (8.5 mmol, 44%). Samples were characterized spectroscopically and compared to references in literature [34]. ^1^H-NMR (δ, CD_3_CN, RT, 400 MHz): 7.76 (m, 2H), 7.59 (m, 2H).

#### 4.1.5. Preparation of Silicon Phthalocyanine Dichloride (via Route C)

Reaction was performed analogously to previously reported literature starting with 1 g of diiminoisoindoline (via Route C) [27]. Crude yield 0.54 g (0.9 mmol, 51%). Samples were characterized spectroscopically and compared to references in literature [33]. HR-MS (EI) for C_32_H_16_Cl_2_N_8_Si [M]^+^: Calcd, 610.0644; Found, 610.0637.

#### 4.1.6. Preparation of Bis(3,4,5-trifluorophenoxy) Silicon Phthalocyanine

Reaction was performed analogous to previously reported literature using as prepared SiPcCl_2_ from Routes A–C. Samples were characterized spectroscopically and compared to references in literature [29]. HR-MS (EI) for C_38_H_18_N_8_OF_3_Si [M–C_6_H_2_OF_3_]^+^: Calcd, 687.1325; Found (Route A), 687.1311; Found (Route B), 687.1330; Found (Route C), 687.1315.

#### 4.1.7. Electrical Testing

Bottom-gate, bottom-contact OTFTs made with (345F)_2_-SiPc active layers were made starting with prefabricated substrates manufactured by Fraunhofer IPMS. These substrates had a doped silicon base/gate, a 230 nm SiO_2_ dielectric, and pre-patterned source-drain electrodes made from gold with an indium tin oxide adhesion layer (W = 2000 µm, L = 2.5, 5, 10, 20 µm). Substrates were rinsed thoroughly with acetone to remove the protective resist and then treated for 15 min in oxygen plasma. Before transferring into a 1% v/v solution of octyltrichlorosilane (OTS, Sigma, 97%) in toluene, the substrates were rinsed with water and isopropanol, then dried with nitrogen. After 60 min heating at 70 °C, the substrates were removed from solution, rinsed with toluene and isopropanol, then dried under vacuum at 70 °C for 30–60 min. Dried, OTS-treated substrates were transferred into a vacuum chamber where (345F)_2_-SiPc was evaporated onto their surface at a rate of 0.3Å/s until their thickness reached the target of 300Å as measured using a quartz crystal monitor. Finished devices were transferred in an evacuated capsule to the custom OTFT testing setup, oesProbe A10000-P290 (Element Instrumentation Inc. & Kreus Design Inc.), and tested under vacuum (P < 0.1 Pa) without having been exposed to air. Transfer characteristics were obtained in the range 0 V < V_GS_ < 60 V, increased step-wise every 100 ms, with V_DS_ set to 50 V. Field-effect mobility (µ_e_) and threshold voltage (V_T_) were calculated from the slope and x-intercept of Equation (1) in the range 35 V < V_GS_ < 45 V, with the dielectric capacitance C_i_ calculated as kɛ_0_/t, where t is the dielectric thickness and k is the relative dielectric constant of SiO_2_ (3.9).

(1) $$I_{\mathrm{DS}}=\frac{{\mu C}_{i}W}{2L}\left(V_{\mathrm{GS}}-V_{T}\right)$$

## Supplementary Materials

The following are available online at https://www.mdpi.com/1996-1944/12/8/1334/s1, Figure S1: Normalized absorbance spectra of (345F)_2_-SiPc via Routes A (black), B (blue) and C (red) in toluene solutions, Figure S2: 1H-NMR spectra of DIII from Route B at 400 MHz in MeCN-d_3_. Spectrum referenced to solvent residual peak at 1.94 ppm, Figure S3: ^1^H-NMR spectra of [H_2_DIII][OTf]_2_ at 300 MHz in MeCN-d_3_. Spectrum referenced to solvent residual peak at 1.94 ppm, Figure S4: ^1^H-NMR spectra of DIII from commercial source (blue) and from Route B (red) in DMSO-d_6_ at 400 MHz. Spectra referenced to residual solvent peak at 2.50 ppm, Figure S5. ^19^F-NMR spectra of [H_2_DIII][OTf]_2_ at 300 MHz in MeCN-d_3_. Spectrum referenced to F_3_CCOH at −76.55 ppm.
