# Supplementary material for: Old Molecule, New Chemistry: Exploring Silicon Phthalocyanines as Emerging N-Type Materials in Organic Electronics
Source: Materials (Basel). 2019 Apr 24;12(8):1334. doi: 10.3390/ma12081334 (PMC6515430; doi:10.3390/ma12081334)
Supplement: Supplementary file 1 [file materials-12-01334-s001.pdf]

materials

Supplementary Materials: Old Molecule, New Chemistry: Exploring Silicon Phthalocyanines as Emerging N-Type Materials in Organic Electronics

Nathan J. Yutronkie, Trevor M. Grant, Owen A. Melville, Benoît H. Lessard and Jaclyn L. Brusso

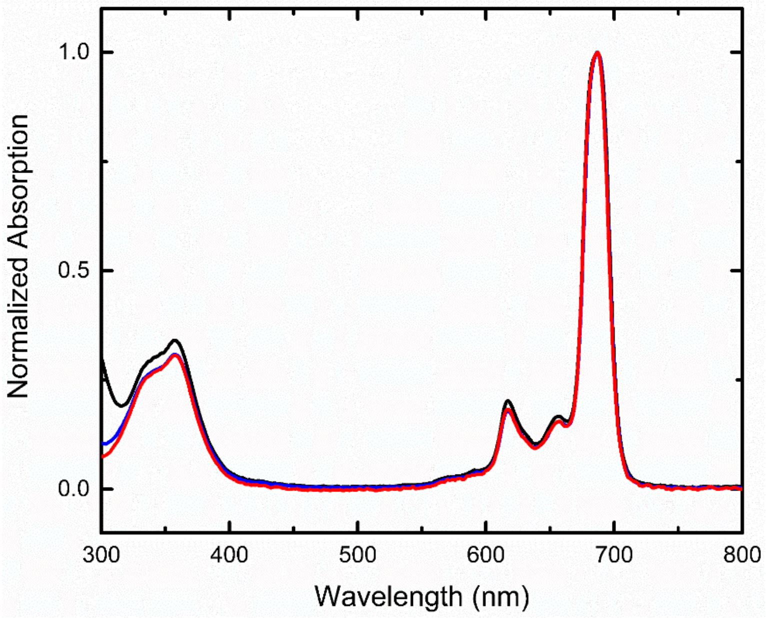

Figure S1. Normalized absorbance spectra of $(345F)_2$-SiPc via Routes A (black), B (blue) and C (red) in toluene solutions.

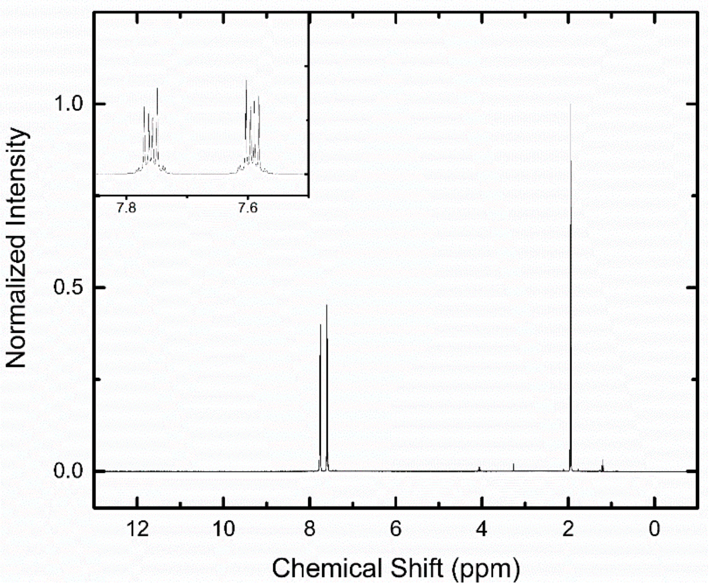

Figure S2. ^{1}H-NMR spectra of DIII from Route B at 400 MHz in MeCN-d_3. Spectrum referenced to solvent residual peak at 1.94 ppm.

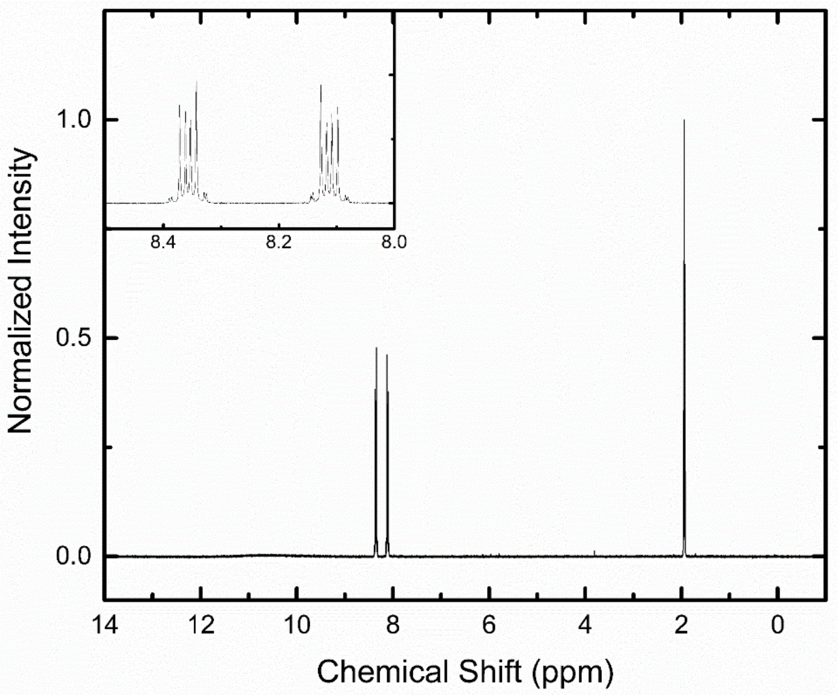

Figure S3. ^{1}H-NMR spectra of $[H_2DIII][OTf]_2$ at 300 MHz in MeCN-d_3. Spectrum referenced to solvent residual peak at 1.94 ppm.

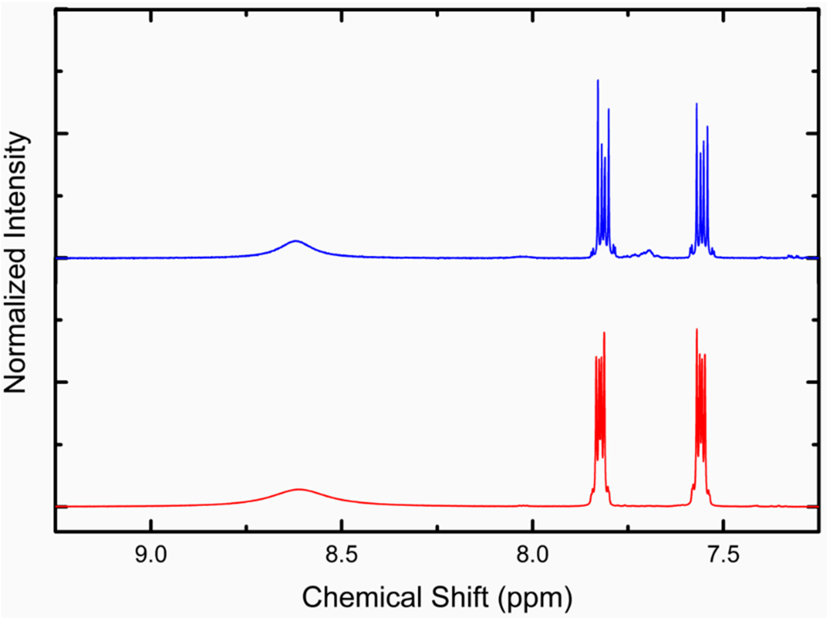

Figure S4. ^{1}H-NMR spectra of DIII from commercial source (blue) and from Route B (red) in DMSO-d_6 at 400 MHz. Spectra referenced to residual solvent peak at 2.50 ppm.

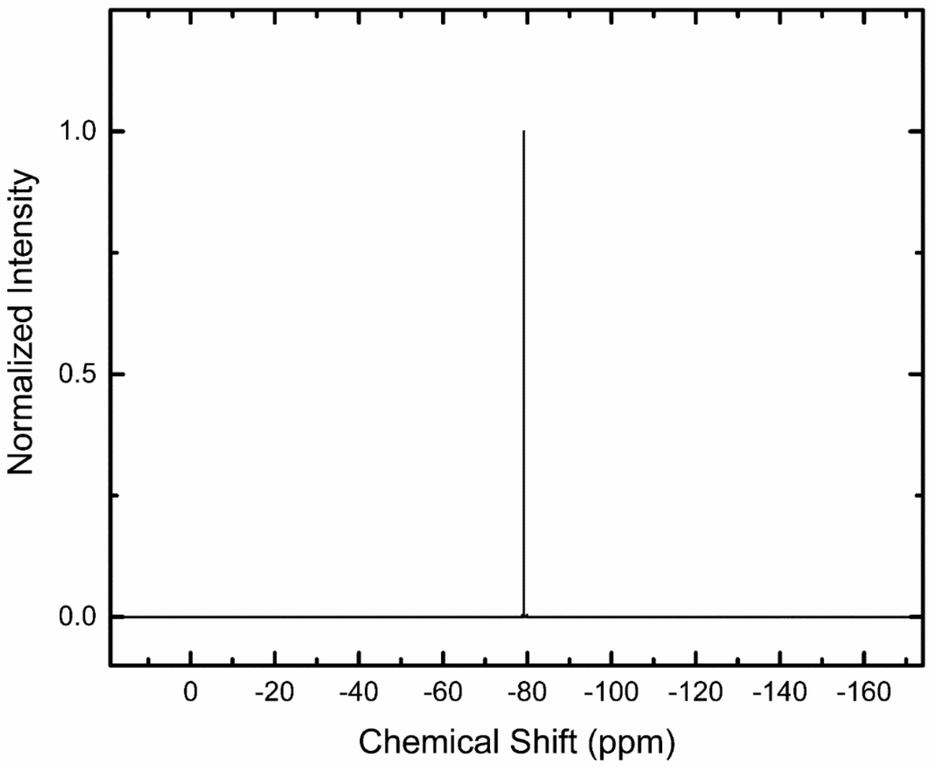

Figure S5. ^{19}F-NMR spectra of $[H_2DIII][OTf]_2$ at 300 MHz in MeCN-d_3. Spectrum referenced to F_3CCOH at −76.55 ppm.

© 2019 by the authors. Submitted for possible open access publication under the terms and conditions of the Creative Commons Attribution (CC BY) license (http://creativecommons.org/licenses/by/4.0/).
